# Postural Orthostatic Tachycardia Syndrome and Its Unusual Presenting Complaints in Women: A Literature Minireview

**DOI:** 10.7759/cureus.2435

**Published:** 2018-04-05

**Authors:** Ibrar Anjum, Wafa Sohail, Betul Hatipoglu, Robert Wilson

**Affiliations:** 1 Internal Medicine, The University of Texas MD Anderson Cancer Center, Houston, TX, Houston, USA; 2 Dow Medical College, Dow University of Health Sciences (DUHS), Karachi, Pakistan; 3 Endocrinology, Cleveland Clinic, Ohio; 4 Neurology, Cleveland Clinic, Ohio

**Keywords:** postural orthostatic tachycardia syndrome, pots and its unusual presentations, pots, postural tachycardia syndrome clinical features, orthostatic intolerance

## Abstract

Postural orthostatic tachycardia syndrome (POTS) is a heterogeneous disorder of the autonomic nervous system that is defined by symptoms of orthostatic intolerance. According to the current criteria for adults, currently, POTS is defined as a heart rate increment of 30 beats/minute or more after 10 minutes of standing in the absence of orthostatic hypotension. There is a vast majority that remains misdiagnosed due to the heterogeneity of the disorder. Due to a lack of Food and Drug Administration (FDA) approved therapy, alternative therapies and over the counter medications are used to alleviate the symptoms. This is an uncommon presentation observed primarily in women, as it is more prevalent in females.

## Introduction and background

Postural orthostatic tachycardia syndrome (POTS) is a disorder of the autonomic nervous system that is defined by symptoms of orthostatic intolerance. It is a final, common pathway of a heterogeneous group of underlying diseases that display similar clinical characteristics and are associated with poor health-related quality of life due to debilitating fatigue, poor sleep, and exercise intolerance [1-3].

Postural orthostatic tachycardia syndrome (POTS) remains to be poorly understood, and many patients suffer without being diagnosed for an extended period. Females of childbearing age predominate this syndrome with a ratio of 5:1. Recent research done helped in understanding some of the pathophysiologies; hence, assisting the physicians to treat the patients more appropriately. But still, there are no FDA approved medicines for POTS, and several medications are used off-label to treat this condition [2].

Postural orthostatic tachycardia syndrome (POTS) affects females more than males [4]. This review article aims to help physicians become familiar with the unique presenting complains of POTS, highlighting the different presenting complaints in women [5]. To serve our purpose, we conducted a PubMed search for at least 50 articles available before 2017.

## Review

According to the current diagnostic criteria, a heart rate increase of 30 beats per minute or more, or above 120 bpm, within the first 10 minutes of standing, in the absence of orthostatic hypotension, is called postural orthostatic tachycardia syndrome (POTS). It should be made clear that POTS and orthostatic intolerance are not similar. Orthostatic intolerance is used to describe a condition in which patients develop symptoms on standing or head uptilt but do not fulfill the criteria for the diagnosis of POTS. In the supine position, about a quarter of body's blood is in the thorax. After assuming an upright posture, there is a roughly 500 cc downward displacement of blood toward the extremities, resulting in a decreased venous return to the heart. In an average person, stabilization occurs in less than one minute. The failure of the body to maintain this aspect of normal hemostasis clinically manifests by varying degrees of hypotension. If the hypotension is significantly profound, it may result in cerebral hypoperfusion, hypoxia, and loss of consciousness [6].

Postural orthostatic tachycardia syndrome (POTS) is the final common pathway of a heterogeneous group of underlying disorders that have similar clinical characteristics. The cluster of symptoms associated with POTS reflects underlying dysautonomia, including palpitation, exercise intolerance, fatigue, lightheadedness, tremor, headache, nausea, syncope, and near syncope [7]. These comprise some of the common presenting complaints of patients with POTS, but many patients present with unusual symptoms, which often lead to misdiagnosis. These include recurrent diarrhea or constipation and bladder problems. Over half of these patients may be seen with the complaint of a migraine headache, intracranial pressure abnormalities, visual disturbances, phonophobia, cognitive impairment, and insomnia [8]. Unintentional weight loss and vagus nerve abnormalities are also associated with the POTS syndrome. Symptoms related to the activation of histamine-producing cells, vascular anomalies, and anhydrosis of lower extremities have also been noticed, as explained below. Although the definition is based on an increased heart rate with orthostatic symptoms while upright, patients with POTS frequently voice complaints beyond what one might attribute to the cardiovascular arena, such as dizziness, lightheadedness, fatigue, etc., apparently reflecting other comorbidities [9-10].

According to Tomljenovic et al.'s case study in 2014, a young school girl presented with flu-like symptoms, such as a sore throat, low-grade fever, fatigue, swollen glands, and intense headaches two months after her human papilloma vaccine (HPV) injection. Her symptoms related to orthostatic intolerance worsened over time so much so that she had to go to school in a wheelchair due to chronic fatigue. After a detailed workup, she was diagnosed with POTS [11]. According to Schondorf et al., 40% of patients with chronic fatigue syndrome also suffer from POTS; indeed, chronic fatigue syndrome (CFS) is frequent and of significant comorbidity in POTS [7].

It was also seen that a majority of patients with postural orthostatic tachycardia syndrome (POTS) also fulfilled criteria for chronic fatigue syndrome (CFS). These symptoms related to CFS were common in POTS patients who did not meet all the criteria for CFS. Symptoms of CFS that overlap in patients with POTS include migraines, disabling fatigue, fibromyalgia, unrefreshing sleep, and impaired memory or concentration

Symptoms of orthostatic intolerance mimicking POTS were found in some patients following bariatric surgery, as proposed by Ponnusamy et al. They described 14 patients with orthostatic intolerance symptoms, including postural dizziness, palpitations, and fainting post-bariatric surgery. They suggested in their retrospective study that the incidence of orthostatic intolerance post-bariatric operation is higher than considered. Detailed autonomic testing conducted by Buchwald et al. in 2004 revealed that 35.7% of such patients fulfilled the criteria for POTS [12]. In a similar study in which all the patients underwent tilt table testing, 20% of patients had a POTS response [13-14]. With the increasing prevalence of obesity and the required clinical interventions, further understanding the pathophysiologic process causing autonomic dysfunction after bariatric interventions will aid in management, which may vary in those with the underlying involvement of the autonomic nervous system, such as diabetes, in whom such procedures are increasingly used [15].

Postural orthostatic tachycardia syndrome (POTS) is also associated with reduced health-related quality of life. It also associated with diabetes, and further research is required due to the increasing prevalence of diabetes in young adults. Sarcoidosis and amyloidosis have also been reported to be related to secondary POTS. A paraneoplastic syndrome associated with adenocarcinoma of the lung, breast, ovary, or pancreas may also present as POTS. These tumors produce autoantibodies that target the acetylcholine receptors in the autonomic ganglia like post viral syndrome [16].

Conner et al. have also reported anhydrosis of the lower extremities. A wide array of dermatologic manifestations are found in association with POTS, which may mislead diagnosis. According to Huang et al., dermatologic manifestations range from livedo reticularis to Raynaud's phenomenon, from cutaneous flushing to erythromelalgia [17]. Mast cell activation and chemical sensitivities were studied for their association with postural orthostatic tachycardia syndrome (POTS) and Ehler Danlos syndrome (EDS) [18]. Facial periodic erythema has also been reported [19]. Connective tissue disorders, especially Ehler Danlos syndrome hypermobility type, have a high concurrence rate with POTS as found by Cohen and Markham [20].

A large proportion of joint hypermobility syndrome patients with esophageal symptoms have exact reflux-related symptoms or esophageal hypomotility, and this is more likely due to POTS [21]. A study was done in 2016 to suggest a potential association between gluten-related disorders and POTS. A prospective study evaluating this relationship further may enable a better understanding and management of these conditions because 4% of patients with POTS had serology and biopsy-proven coeliac disease in that study, which was significantly higher in the coeliac disease population than in the local population [22].

In recent years, research on POTS has shifted toward work on renin–angiotensin–aldosterone system (RAAS) [23]. Patients with this syndrome have a partial neuropathic state with impaired lower extremity sympathetic innervation, abnormal venous pooling, and hypovolemic state with low renin–angiotensin–aldosterone system upregulation [24]. A study showed that some patients with POTS had inappropriately normal levels of plasma renin activity and paradoxically lower levels of aldosterone despite their hypovolemia when compared to healthy subjects [25].

Sleep disturbance is an essential factor in the quality of life. Many chronically ill patients, including those with POTS, complain of poor sleep but a lack of sufficient data to link between POTS, depression, and disturbed sleep might have limited the variability, as explained by Pederson and Book [26]. Sleep issues were adequately treated through pharmacologic and nonpharmacologic means [27-28].

 As we know from the underlying pathophysiology of postural orthostatic tachycardia syndrome (POTS), that it is an autonomic dysfunction, it would likely affect the lower urinary tract functions as well. However, a precise relationship between bladder function and autonomic dysfunction have not been established [29]. Some studies have been conducted to deduce a defining relationship, but they are at an initial level [30]. One thing worth mentioning here is that with the help of these studies, a possible association between idiopathic overactive bladder and autonomic dysfunction has been established [31].

We are now aware of the fact that POTS predominates in females; its presentation with other diseases in women is being studied to find out a potential relationship. Fibromyalgia, which is also a disease of young women, was found to be associated with POTS in a study [32-33]. Other manifestations of orthostatic intolerance, such as chronic fatigue syndrome and sleep disturbance, also seem to have some relationship.

Moon et al.'s study evaluated how the diagnosis of POTS can be missed in a single orthostatic stress test because orthostatic tachycardia is more prominent in the morning, but not always. Therefore, they suggest repeated orthostatic stress tests in clinically suspected postural orthostatic tachycardia syndrome patients [34]. A case report published in 2016 described two patients who were diagnosed with POTS after all the necessary tests were done. The first patient's symptoms decreased without any medication. However, in the second patient, different drugs were administered, but only ivabradine had beneficial effects on the patient's symptoms [35]. Several factors contribute to difficulties in the management of POTS. First is the lack of patient information or patient misinformation about the disorder. This leads to unrealistic expectations about the beneficial effects of treatment aimed to correct the postural tachycardia and to secondary frustration and symptom amplification.This indicates that further research on the unusual presentation of patients with postural orthostatic tachycardia syndrome is required.

## Conclusions

Due to the heterogeneous nature of postural orthostatic tachycardia syndrome (POTS) symptoms and its frequent co-occurrence with other systemic diseases, POTS is challenging to diagnose. We have tried to present unusual manifestations of this syndrome by conducting a thorough review of the literature available online. There is no single treatment modality for POTS. With this literature review, we have tried to highlight the unusual presenting complains of POTS, which may help in keeping POTS as an important differential diagnosis in outpatient clinics. This will assist in devising proper treatment modalities by conducting further research on this critical topic.
